# Supplementary material for: Decision-making in livestock biosecurity practices amidst environmental and social uncertainty: Evidence from an experimental game
Source: PLoS One. 2019 Apr 17;14(4):e0214500. doi: 10.1371/journal.pone.0214500 (PMC6469775; doi:10.1371/journal.pone.0214500)
Supplement: S3 File — (DOCX) [file pone.0214500.s003.docx]

### S3 Data Captions

Data are stored in the csv file titled “S2_Dataset".

The structure of this file is as follows:

'data.frame': 1979 obs. of 12 variables:

$ pctMaxLstDcnMnth : numeric 0 0 0.667 0 0.667 ...

$ Player : numeric 1 1 1 1 1 1 1 1 1 1 ...

$ LastDecnMnth : numeric 11 7 8 11 5 11 11 11 3 7 ...

$ probVisibleLastMonth : numeric 0 0.0283 0.2997 0 0.0386 ...

$ ProbUnknownLastDecsn : numeric m 0.1369 0.041 0.0505 0.115 0.0212 ...

$ DiseaseVis : catergorical 1 2 2 2 2 2 2 3 1 3 ...

$ BiosecurityVis : catergorical 3 3 2 3 1 2 1 3 3 1 ...

$ trtIndex : catergorical 13 23 22 23 21 22 21 33 13 31 ...

$ meanBiosecurityVisible: numeric 2.51 0.469 0.533 2.469 0 ...

$ SessionNumber : numeric 3 4 5 6 7 8 9 10 11 12 ...

$ timeSinceHit : numeric 1 2 1 1 2 3 4 5 6 7 ...

$ knownAndUnknownProb : numeric 0.1369 0.0682 0.335 0.115 0.0589 ...

where column headers correlate to the data in the manuscript as detailed in S1 Table.

**S1 Table. Data Captions**

| **Manuscript data label and details** | **Csv column header and data name** |
| --- | --- |
| Percent Maximum Biosecurity (PMB. The dependent variable) | pctMaxLstDcnMnth |
| Participant (Random variable) | Player |
| Last Decision Month (LM) | LastDecnMnth |
| Observed Probability of Infection (PI) The probability of acquiring an infection in the next round based on information visible to the participant. This probability is for the last month where the participant was able to make a decision. | probVisibleLastMonth |
| The probability of acquiring an infection in the next round based on information that was not visible to the participant. This probability is for the last month where the participant was able to make a decision. | ProbUnknownLastDecsn |
| Environmental Uncertainty Treatment (EUT). Information sharing of disease incidence in the simulation-controlled facilities. Disease Visibility Treatment. This is a categorical variable. 1: no computer simulated agent disease information was visible to the participant. 2: 15 out of 49 computer simulated agents’ disease information was visible to the participant. 3: all (49 out of 49) computer simulated agents’ disease information was visible to the participant. | DiseaseVis |
| Social Uncertainty Treatment (SUT). Social Uncertainty: Information sharing of biosecurity levels in the simulation-controlled facilities. Biosecurity Visibility Treatment. This is a categorical variable. 1: no computer simulated agent biosecurity information was visible to the participant. 2: 15 out of 49 computer simulated agents’ biosecurity information was visible to the participant. 3: all (49 out of 49) computer simulated agents’ biosecurity information was visible to the participant. | BiosecurityVis |
| Treatment Interaction term. Unused in manuscript. | trtIndex |
| Observed Biosecurity Level (OBL). Level of observed biosecurity reported by simulation-controlled facilities. Type 1 biosecurity is generally high biosecurity with a mean of 2.53 and Type 2 biosecurity is generally low biosecurity with a mean observed value of 0.46 | meanBiosecurityVisible |
